# The influence of selected microRNAs on the expression profile of genes and proteins related to the tumor necrosis factor-alpha signaling pathways in endometrioid endometrial cancer

**DOI:** 10.1007/s00432-023-04863-3

**Published:** 2023-05-26

**Authors:** Nikola Zmarzły, Szymon Januszyk, Paweł Mieszczański, Justyna Czarniecka, Anna Bednarska-Czerwińska, Dariusz Boroń, Marcin Oplawski, Beniamin Oskar Grabarek

**Affiliations:** 1 Department of Histology, Cytophysiology and Embryology, Faculty of Medicine in Zabrze, Academy of Silesia in Katowice, 41-800 Zabrze, Poland; 2 Department of Gynecology and Obstetrics, Faculty of Medicine in Zabrze, Academy of Silesia in Katowice, 41-800 Zabrze, Poland; 3 ICZ Healthcare Hospital in Zywiec, 34-300 Zywiec, Poland; 4 Hospital of Ministry of Interior and Administration, 40-052 Katowice, Poland; 5 Gyncentrum, Laboratory of Molecular Biology and Virology, 40-851 Katowice, Poland; 6 American Medical Clinic, 40-600 Katowice, Poland; 7 Department of Gynecology and Obstetrics with Gynecologic Oncology, Ludwik Rydygier Memorial Specialized Hospital, 31-826 Kraków, Poland; 8 Department of Gynecology and Obstetrics, TOMMED Specjalisci od Zdrowia, 40-662 Katowice, Poland; 9 Department of Gynecology and Obstetrics, Faculty of Medicine and Health Sciences, Andrzej Frycz Modrzewski University in Cracow, 30-705 Cracow, Poland

**Keywords:** Endometrial cancer, TNF-α, miRNA, TNF signaling, Expression profile

## Abstract

**Purpose:**

Tumor necrosis factor exerts many adverse biological effects, from cell proliferation to cell death. Accurate diagnosis and treatment are therefore difficult due to many factors influencing tumor necrosis factor-alpha (TNF-α) signaling, including microRNAs (miRNAs), especially in tumors. The aim of the study was to determine the influence of miRNAs on the expression profile of genes and proteins related to TNF-α signaling in endometrial cancer.

**Methods:**

The material consisted of 45 endometrioid endometrial cancer and 45 normal endometrium tissue samples. Gene expression was determined with microarrays and then validated for TNF-α, tumor necrosis factor receptor 1 (TNFR1) and 2 (TNFR2), caveolin 1 (CAV1), nuclear factor kappa B subunit 1 (NFKB1), and TGF-beta activated kinase 1 (MAP3K7)-binding protein 2 (TAB2) using real-time quantitative reverse transcription reaction (RT-qPCR). The protein concentration was assessed by enzyme-linked immunosorbent assay (ELISA). In addition, differentiating miRNAs were identified using miRNA microarrays and their relationships with TNF-α signaling genes were evaluated using the mirDIP tool.

**Results:**

TNF-α, TNFR1, TNFR2, CAV1, NFKB1, and TAB2 were upregulated both on the mRNA and protein levels. The decrease in the activity of miR-1207-5p, miR-1910-3p, and miR-940 may be related to CAV1 overexpression. Similarly for miR-572 and NFKB1 as well as miR-939-5p and TNF-α. In turn, miR-3178 may partially inhibit TNFR1 activity up to grade 2 cancer.

**Conclusion:**

TNF-α signaling, especially the TNF-α/NF-κB axis, is disrupted in endometrial cancer and worsens with disease progression. The observed changes may be the result of miRNAs’ activity in the initial stage of endometrial cancer and its gradual loss in later grades.

## Introduction

Endometrial cancer is one of the most commonly diagnosed gynecological cancer worldwide (Sung et al. 2021). It is diagnosed primarily in postmenopausal women, although up to 25% of cases occur in premenopausal women (Rizzo et al. 2018). Over the years, various classification systems have been developed combining histological features, including grading, as well as genetic features such as DNA polymerase epsilon catalytic subunit (POLE) subtypes (Boroń et al. 2021). Depending on the genetic characteristics, four subgroups have been identified: POLE-ultramutated, microsatellite instability-hypermutated, copy-number low, and copy-number high (Yu et al. 2019). However, this classification is difficult to include in routine diagnostics, as it is associated with technical difficulties and high costs. As an alternative, the Proactive Molecular Risk Classifier for Endometrial Cancer (ProMisE) has emerged, where immunohistochemical markers have been proposed instead of sequencing. However, markers for all The Cancer Genome Atlas (TCGA) molecular groups have not yet been found (Raffone et al. 2021). The best solution seems to be to combine several systems to get the best picture of the course of the disease.

Tumor necrosis factor-alpha (TNF-α) is a pleiotropic cytokine that is largely involved in inflammation and the immune response. Its activity is possible due to its binding to tumor necrosis factor receptor 1 (TNFR1) and 2 (TNFR2), which results in the activation of numerous signaling cascades (Wajant 2009). Depending on the signal strength and the molecules involved, TNFR1 signal transduction can induce apoptosis or cell survival. In turn, interaction with TNFR2 mainly involves cell activation, proliferation, and migration (Mercogliano et al. 2020). Thus, TNF-α signaling can suppress tumorigenesis as well as promote angiogenesis, migration, and invasion of tumor cells (Zhao and Zhang 2018). TNF-α is considered an attractive therapeutic target, especially since TNF-α inhibitors, such as monoclonal antibodies and fusion proteins, are already used to treat autoimmune and inflammatory diseases (Mercogliano et al. 2020). However, numerous relationships within TNF-α signaling and differences depending on the stage and type of tumor hinder the development of precise diagnostics and treatment. Moreover, apart from the intertwining of signaling pathways, it is also worth paying attention to the participation of miRNAs in the regulation of gene activity. These molecules affect the activity of many genes, including NF-κB, which is involved in proliferation and tumor development, and is also an inflammation initiator and TNF-α downstream target (Koeck et al. 2018). Inhibition of the TNF-α/NF-κB axis by miRNAs may attenuate the invasive potential of cancer cells, as shown in colorectal cancer (Shen et al. 2017). On the other hand, high levels of miRNAs may also promote excessive activation of NF-κB and, as a result, promote cancer aggressiveness (Zhao et al. 2021a). TNF-α can induce the expression of miRNAs, which leads to increased cell motility and thus enhanced tumor invasiveness by activating extracellular signal-regulated kinase 1/2 (ERK1/2) signaling pathway (Hsing et al. 2019). Interestingly, miRNAs can directly modulate the activity of TNF-α ligands and adapter molecules, which affects cell survival. They may also influence the induction of apoptosis by participating in the formation of the receptor-interacting serine/threonine-protein kinase 1 (RIP1), FAS-associated death domain protein (FADD), and caspase 8 complex (Chakraborty et al. 2020).

The aim of the study was to determine the influence of miRNAs on the expression profile of genes and proteins related to TNF-α signaling.

## Results

### Expression profile of genes associated with TNF-α signaling determined by mRNA microarrays

**Fig. 1:**
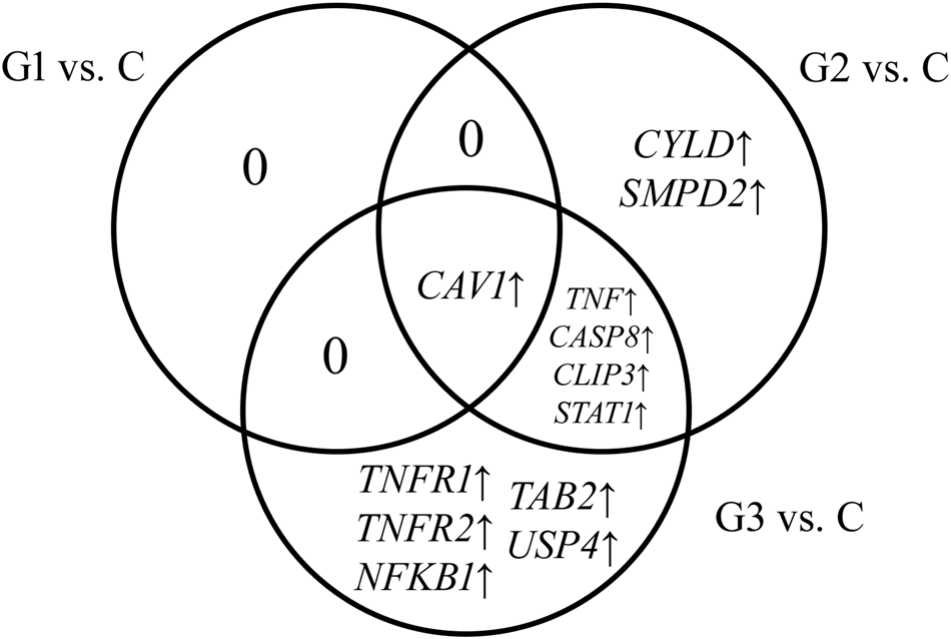
Venn diagram showing the distribution of genes related to TNF-α signaling in endometrial cancer

Among 106 mRNAs corresponding to genes related to TNF-α signaling, significant expression changes were noted for 14 mRNAs, including 2 for G1, 8 for G2, and 11 for G3. The construction of the Venn diagram revealed which mRNAs are characteristic of a given grade and which are common (Fig. 1).

In the case of G1 cancer, no characteristic mRNA was found. *CYLD *and *SMPD2* were characteristics of G2 cancer, and *TNFR1*, *TNFR2*, *NFKB1*, *TAB2*, and *USP4* for G3 cancer. It was also observed that *TNF-α*, *CASP8*, *CLIP3*, and *STAT1* are characteristics of G2 and G3 cancer. Moreover, *CAV1* was a differentiating gene regardless of cancer grade. Table 1 lists the detailed fold-change values for each grade of endometrial cancer.

**Table 1:** List of TNF-α signaling transcripts differentiating endometrial cancer from control (*p* < 0.05 and FC > 2 or FC <  − 2)

ID	mRNA	G1 vs. C	G2 vs. C	G3 vs. C
213295_at	*CYLD*	− 1.60	− 2.42*	− 1.60
205622_at	*SMPD2*	1.35	2.05*	1.85
200887_s_at	*STAT1*	1.32	2.27*	1.53
207643_s_at	*TNFR1*	1.20	1.60	2.43*
203508_at	*TNFR2*	1.08	1.54	2.19*
209239_at	*NFKB1*	1.23	2.53	2.71*
209969_s_at	*STAT1*	1.19	1.11	2.12*
210284_s_at	*TAB2*	1.09	1.31	2.75*
202682_s_at	*USP4*	1.47	− 1.04	2.54*
213373_s_at	*CASP8*	1.36	2.33*	2.09*
212358_at	*CLIP3*	− 1.52	− 5.08*	− 2.34*
207113_s_at	*TNF-α*	1.24	2.51*	3.07*
203065_s_at	*CAV1*	4.17*	11.63*	12.64*
212097_at	*CAV1*	5.10*	18.57*	11.09*

*CYLD* cylindromatosis tumor suppressor, *SMPD2* sphingomyelin phosphodiesterase 2, *STAT1* signal transducer and activator of transcription 1, *TNF-α* tumor necrosis factor-alpha, *TNFR1* tumor necrosis factor receptor 1, *TNFR2* tumor necrosis factor receptor 2, *CAV1* caveolin 1, *NFKB1* nuclear factor kappa B subunit 1, *TAB2* TGF-beta activated kinase 1 (MAP3K7) binding protein 2, *USP4* ubiquitin specific peptidase 4, *CASP8* caspase 8, *CLIP3* CAP-Gly domain-containing linker protein 3, *ID*, number of the probe, *FC* fold-change, *C* control, *G* grade of endometrial cancer

*p < 0.05 vs. C group

Overexpression in all cancer grades was observed for *SMPD2*, *STAT1*, *TNF-α*, *TNFR1*, *TNFR2*, *NFKB1*, *TAB2*, *CASP8*, and *CAV1*. The greatest changes were noted for *CAV1*, where the FC value in G2 and G3 cancers exceeded 10. Moreover, expression in G2 cancer increased about threefold compared to G1 cancer. In turn, a decrease in expression was noted for *CYLD* and *CLIP3* in all cancer grades. *USP4* level was elevated in G1 and G3, while it was decreased in G2 cancer. Changes in the expression of the studied genes increase with the progression of endometrial cancer.

### Expression profile of TNF-α, TNFR1, TNFR2, CAV1, NFKB1, and TAB2 at the mRNA and protein level determined by RT-qPCR and ELISA

The Shapiro–Wilk test revealed that the obtained results do not meet the assumptions of the normal distribution. Next, the Kruskal–Wallis and Dunn’s tests showed statistically significant differences in the expression of all tested genes. Table 2 shows the median, first (Q1) and third (Q3) quartiles, and the results of the statistical analysis.

**Table 2:** Values of descriptive statistics, Kruskal–Wallis, and Dunn’s post hoc tests in endometrial cancer and control (*p* < 0.05)

Gene	Group	mRNA copies/μg total RNA			Kruskal–Wallis test	Dunn’s post hoc test
		Me	Q1	Q3		
*TNF-α*	C	184,080	160,500	226,010	< 0.001	G1 vs. C, *p* = 0.002 G2 vs. C, *p* < 0.001 G3 vs. C, *p* < 0.001 G3 vs. G2, *p* = 0.003 G3 vs. G1, *p* < 0.001 G2 vs. G1, *p* = 0.003
	G1	370,650	330,400	446,700		
	G2	597,000	577,060	678,200		
	G3	991,600	942,300	1,098,000		
*TNFR1*	C	267,330	236,060	286,510	< 0.001	G1 vs. C, *p* < 0.001 G2 vs. C, *p* < 0.001 G3 vs. C, *p* < 0.001 G3 vs. G2, *p* < 0.001 G3 vs. G1, *p* < 0.001 G2 vs. G1, *p* = 0.223
	G1	583,080	497,010	630,520		
	G2	623,920	567,920	701,690		
	G3	994,940	890,760	1,152,900		
*TNFR2*	C	246,140	225,260	274,140	< 0.001	G1 vs. C, *p* = 0.010 G2 vs. C, *p* < 0.001 G3 vs. C, *p* < 0.001 G3 vs. G2, *p* = 0.644 G3 vs. G1, *p* < 0.001 G2 vs. G1, *p* < 0.001
	G1	360,260	324,390	442,300		
	G2	593,120	564,240	658,290		
	G3	624,320	546,510	731,490		
*CAV1*	C	47,000	36,900	60,120	< 0.001	G1 vs. C, *p* = 0.003 G2 vs. C, *p* < 0.001 G3 vs. C, *p* < 0.001 G3 vs. G2, *p* < 0.001 G3 vs. G1, *p* = 0.066 G2 vs. G1, *p* < 0.001
	G1	172,850	163,020	185,800		
	G2	1,054,360	908,790	1,250,260		
	G3	796,170	724,140	901,950		
*NFKB1*	C	49,140	46,430	56,670	< 0.001	G1 vs. C, *p* = 0.003 G2 vs. C, *p* < 0.001 G3 vs. C, *p* < 0.001 G3 vs. G2, *p* = 0.021 G3 vs. G1, *p* < 0.001 G2 vs. G1, *p* = 0.001
	G1	73,240	69,030	79,520		
	G2	91,010	88,420	96,230		
	G3	99,870	97,840	105,670		
*TAB2*	C	17,140	16,010	18,010	< 0.001	G1 vs. C, *p* = 0.016 G2 vs. C, *p* < 0.001 G3 vs. C, *p* < 0.001 G3 vs. G2, *p* = 0.020 G3 vs. G1, *p* < 0.001 G2 vs. G1, *p* < 0.001
	G1	20,650	19,490	22,640		
	G2	27,780	27,120	32,300		
	G3	36,240	32,510	38,940		

*TNF-α* tumor necrosis factor-alpha, *TNFR1* tumor necrosis factor receptor 1, *TNFR2* tumor necrosis factor receptor 2, *CAV1* caveolin 1, *NFKB1* nuclear factor kappa B subunit 1, *TAB2* TGF-beta activated kinase 1 (MAP3K7)-binding protein 2, *Me* median, *Q1* lower quartile, *Q3* upper quartile, *C* control, *G* grade of endometrial cancer

The obtained RT-qPCR results revealed the overexpression of all studied genes, which is consistent with the results of the microarray experiment. Their elevated level was significant in each cancer grade compared to the control. In addition, for *TNF-α*, *NFKB1*, and *TAB2*, all comparisons within endometrial cancer grades were also significant.

As part of the result validation, the concentration of *TNF-α*, *TNFR1*, *TNFR2*, *CAV1*, *NFKB1*, and *TAB2* was also assessed at the protein level (Table 3).

**Table 3:** Concentration of TNF-α, TNFR1, TNFR2, CAV1, NFKB1, and *TAB2* in the study and control group (*p* < 0.05)

Group	C	G1	G2	G3
TNF-α	12.36 ± 1.08	19.15 ± 4.18	33.58 ± 10.40*	59.62 ± 9.65*
TNFR1	16.55 ± 4.71	21.99 ± 8.51	40.13 ± 13.70*	64.03 ± 22.80*
TNFR2	15.80 ± 4.90	23.90 ± 6.20	36.74 ± 9.45*	60.93 ± 10.90*
CAV1	20.31 ± 4.62	40.96 ± 14.80	76.11 ± 20.00*	117.34 ± 25.60*
NFKB1	94.81 ± 8.74	94.86 ± 6.89	120.21 ± 10.50*	134.44 ± 9.10*
TAB2	48.31 ± 10.40	65.76 ± 9.94	80.71 ± 9.31*	123.37 ± 14.70*

*TNF-α* tumor necrosis factor-alpha, *TNFR1* tumor necrosis factor receptor 1, *TNFR2* tumor necrosis factor receptor 2, *CAV1* caveolin 1, *NFKB1* nuclear factor kappa B subunit 1, *TAB2* TGF-beta activated kinase 1 (MAP3K7)-binding protein 2, *C* control, *G* grade of endometrial cancer

*p < 0.05 vs. C group

The obtained results show the overexpression of the studied genes in endometrial cancer compared to the control, which is consistent with the analysis at the mRNA level. Significant changes were noted for G2 and G3 samples.

### Overall survival analysis

**Fig. 2:**
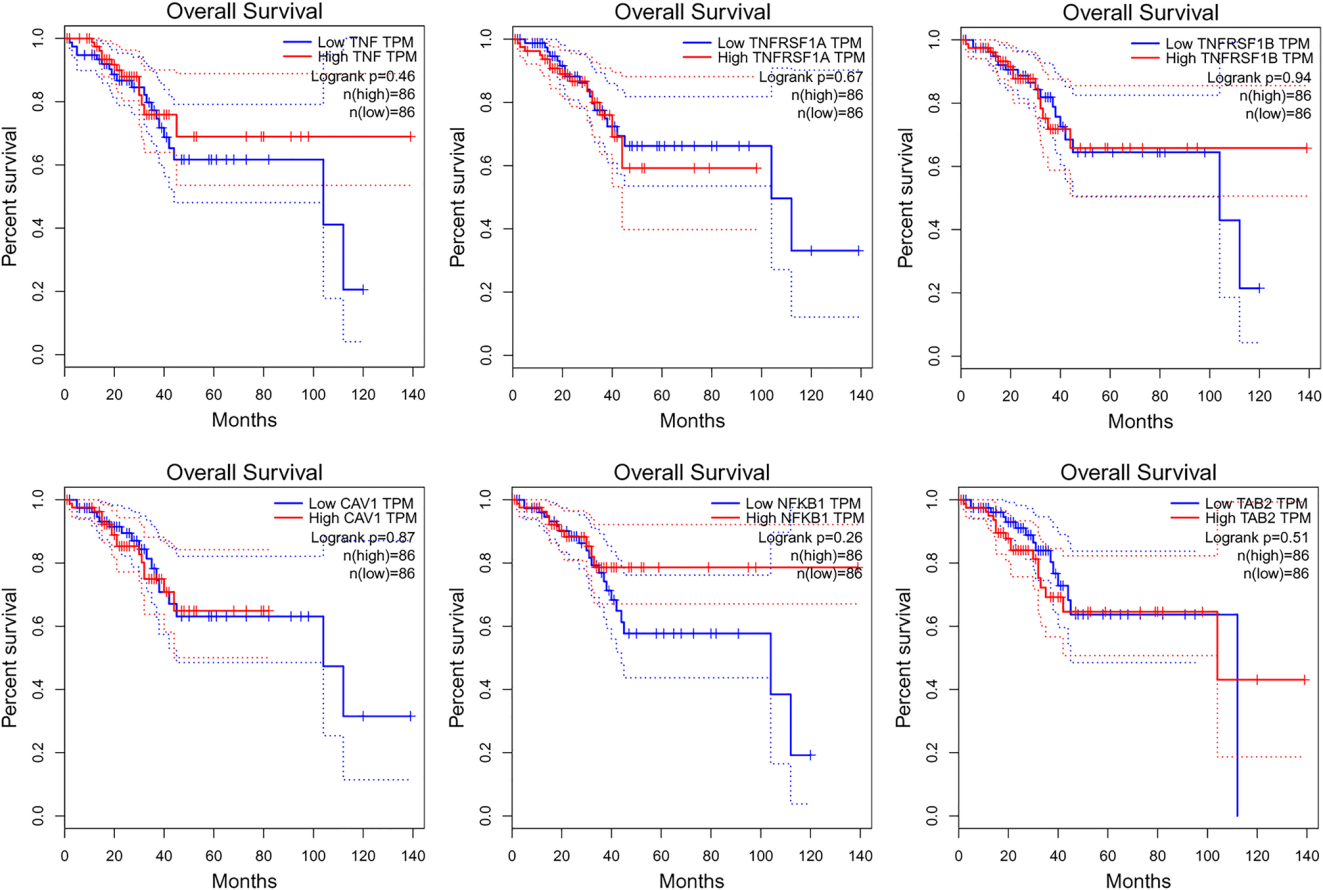
Overall survival analysis for *TNF-α*, *TNFR1*, *TNFR2*, *CAV1*, *NFKB1*, and *TAB2* based on the GEPIA database

Overall survival (OS) analysis based on the GEPIA database was performed for the studied genes. The results are shown in Fig. 2.

The analysis showed a similar OS result for both the high and low levels of each gene group. From about 40 months, better OS was noted for high-*TNF*, low-*TNFR1*, and high-*NFKB1*. In the case of *TNFR2*, worse OS was observed for low-*TNFR2* only after 100 months, and similarly for *CAV1* and *TAB2*.

Using the GEPIA database, it was also noted that the studied genes do not belong to the top 100 most differential survival genes for both overall survival and disease-free survival.

### miRNA target prediction

Among 1100 miRNAs that can be found on a microarray plate, a significant change in expression was reported for 178 miRNAs. Further analysis revealed that 131 miRNAs differentiate G1, 58 miRNAs differentiate G2, and 84 miRNAs differentiate G3 from the control. 76 miRNAs were characteristics of G1 cancer, 6 miRNAs of G2 cancer, and 24 miRNAs of G3 cancer. 23 miRNAs were differentiating regardless of cancer grade. 12 miRNAs were common to G1 and G2, 17 miRNAs to G2 and G3, and 20 miRNAs to G3 and G1 cancers. The list of these 178 differentiating miRNAs was uploaded to the mirDIP database and predictions were made which of them may participate in the regulation of *TNF*, *TNFR1*, *TNFR2*, *CAV1*, *NFKB1*, and *TAB2* gene expression (Table 4).

**Table 4:** List of TNF-α signaling-related genes whose activity may be regulated by miRNAs in endometrial cancer

mRNA	Expression	miRNA	FC		
			G1	G2	G3
*CAV1*	Increased	miR-1207-5p	12.25*	1.57	1.03
		miR-1910-3p	12.77*	1.79	− 2.82
		miR-940	2.4*	1.31	− 1.03
*NFKB1*	Increased	miR-572	11.84*	− 1.41	− 1.46
*TAB2*	Increased	miR-1228-3p	3.36*	1.57	− 3.47*
		miR-134-3p	− 3.15	− 6.02*	− 16.07*
		miR-143-5p	− 4.07*	− 4.11*	− 4.67*
		miR-155-5p	− 10.99*	3.00*	1.21
		miR-22-5p	− 27.27*	− 3.28	− 5.37
		miR-29a-5p	− 23.02*	− 4.42	− 1.71
*TNF-α*	Increased	miR-939-5p	6.55*	1.46	− 1.45
*TNFR1*	Increased	miR-3178	3.38*	5.52*	− 1.15
*TNFR2*	Increased	miR-769-3p	1.13	1.44	4.95*

*CAV1* caveolin 1, *NFKB1* nuclear factor kappa B subunit 1, *TAB2* TGF-beta activated kinase 1 (MAP3K7)-binding protein 2, *TNF-α* tumor necrosis factor-alpha, *TNFR1* tumor necrosis factor receptor 1, *TNFR2* tumor necrosis factor receptor 2, *FC* fold-change, *C* control, *G* grade of endometrial cancer

*p < 0.05 vs. C group

The obtained results indicate that miR-1207-5p, miR-1910-3p, and miR-940, the activity of which decreases with tumor progression, may be involved in the regulation of *CAV1* expression. Similarly for *NFKB1* and *TNF-α* which may be influenced by miR-572 and miR-939-5p, respectively. It has been observed that the *TAB2* level can be affected by six miRNAs, including miR-1228-3p, miR-134-3p, miR-143-5p, miR-155-5p, miR-22-5p, and miR-29a-5p, which mostly show reduced expression. A level decrease was also noted for miR-3178 whose target may be *TNFR1* as opposed to miR-769-3p matched with *TNFR2*, whose expression increases with cancer grade.

**Fig. 3:**
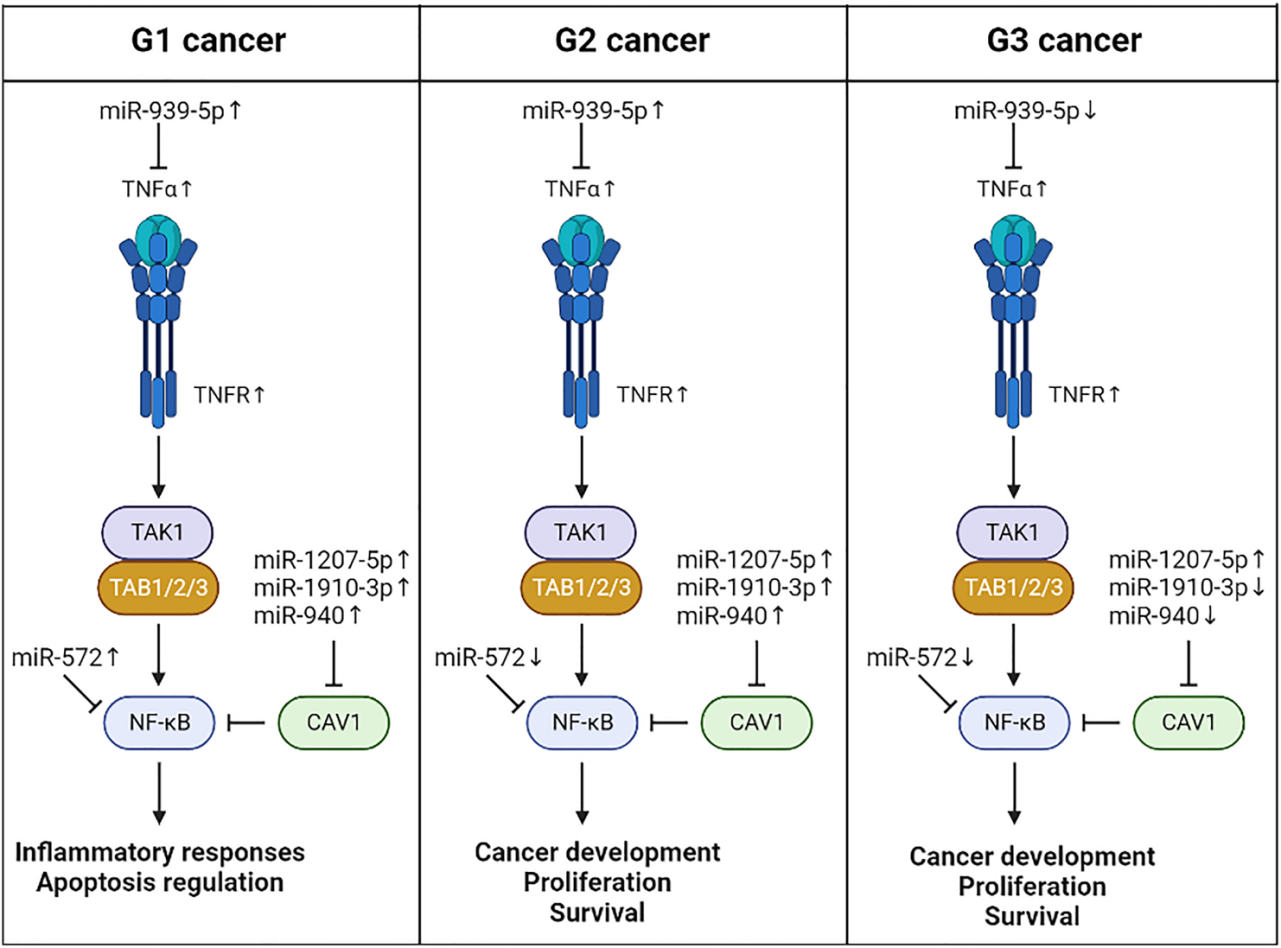
Main results regarding TNF-α signaling in endometrial cancer. The arrows show the direction of changes in the expression of TNF-α, its receptors, and miRNAs involved in the regulation of TNF-α signaling in individual grades of endometrial cancer in relation to the control

In summary, the main results regarding TNF-α signaling are presented in Fig. 3.

## Discussion

As part of this study, an analysis of the expression profile was performed, both at the mRNA and protein levels, in endometrial cancer and control. In addition, miRNAs differentiating particular cancer grades were identified followed by prediction which of them may regulate the activity of selected genes involved in TNF-α signaling. Multilevel analysis allows to fully trace the flow of genetic information, which translates into a better understanding of the observed phenomena.

TNF-α exerts many biological effects, from cell proliferation to cell death, and is involved in tumor initiation and progression, including gastric cancer (Oshima et al. 2014) and ovarian cancer (Lau et al. 2017). What effect will be triggered depends on the interaction between signaling pathways, the type of receptor TNF-α binds to, the type of adapter proteins or the interaction with miRNAs. In our study, TNF-α and both receptors showed a significant increase in expression compared to the control. Smith et al. also recorded TNF-α levels in endometrial cancer (Smith et al. 2013). Our analysis indicated that TNF-α is a target for miR-939-5p, showing the greatest activity in G1 cancer, which then gradually declines. Shen et al. found miR-939-5p to be involved in pancreatic cancer migration and invasion (Shen et al. 2020). In turn, Zhao et al. observed inhibition of the aggressive osteosarcoma phenotype by this miRNA (Zhao et al. 2019). In the case of TNFR1, its expression can be regulated by miR-3178, which activity increases until G2 cancer and then declines. Wu et al. observed that low levels of miR-3178 in gastric cancer favored the proliferation and migration of neoplastic cells (Wu et al. 2022). Similar conclusions were presented by Kong et al. in the triple-negative breast cancer (Kong et al. 2018). In turn, expression of TNFR2 could potentially be regulated by miR-769-3p, whose expression increased with cancer grade. However, given the increase in TNFR2 level in cancer samples compared to control at both mRNA and protein levels, it is possible that this miRNA does not participate in its regulation in endometrial cancer.

Caveolin 1 (CAV1) belongs to the family of structural proteins involved in caveolae formation, which are regulators of signal transduction. Interestingly, caveolin 1 deficiency is associated with premature cell aging due to mitochondrial dysfunction (Yu et al. 2017). The biological context is of great importance as CAV1 can both favor cancer progression and its inhibition due to participation in such processes as apoptosis, invasion, or migration (Williams and Lisanti 2005). In breast cancer, CAV1 activity has been shown to be associated with resistance to radio- and chemotherapy (Qian et al. 2019). High CAV1 levels have been reported in pancreatic cancer (Demirci et al. 2017), while decreased CAV1 levels were observed in colorectal cancer (Torrejón et al. 2017) and hepatocellular carcinoma (Tang et al. 2012). Furthermore, Yang et al. noticed that a high CAV1 level reduces the metastatic potential of colon cells (line SW480) and may indicate a promising outcome in patients with this cancer (Yang et al. 2018). In endometrial cancer, Diaz-Valdivia et al. recorded a high CAV1 level that favored its progression (Diaz-Valdivia et al. 2015), which is consistent with our results. In our study, elevated CAV1 expression was confirmed at the mRNA and protein levels. Moreover, the concentration of CAV1 progressing with cancer grade may be related to the decreasing activity of miR-1207-5p, miR-1910-3p, and miR-940. Chen et al. confirmed that miR-1207-5p is a gastric cancer suppressor and can be used in therapy (Chen et al. 2014). Similar conclusions were drawn by Dang et al. in the lung cancer metastasis study (Dang et al. 2016) and by Wu et al. for laryngeal squamous cell carcinoma (Wu et al. 2021). MiR-1910-3p is considered a progression suppressor of the esophageal squamous cell carcinoma (Meng et al. 2018) and prostate cancer (Xu et al. 2021a). In addition, Wang et al. noted that this miRNA promotes breast cancer metastasis by activating NF-κB signaling (Wang et al. 2020). In turn, Rajendiran et al. indicated miR-940 as a potential prostate cancer biomarker (Rajendiran et al. 2021), while Ma et al. showed its protective effect in the early stage of breast cancer, which results from targeting, inter alia, TNF-α signaling (Ma et al. 2021). The decrease in the level of these miRNAs with the grade of cancer with a simultaneous increase in CAV1 level may indicate that the progression of endometrial cancer is accompanied by a loss of the protective effect of these miRNAs.

We drew similar conclusions for NF-κB as we noticed that its expression increases with cancer grade. In addition, it was a gene characteristic of G3 cancer. Interestingly, miR-572 for which NFKB1 is the target showed significant overexpression in G1 cancer followed by a sharp decline in the remaining grades. Disturbances in the activity of the NF-κB family in endometrial cancer have previously been described by Pallares et al. (Pallares et al. 2004). NF-κB participates in the regulation of the activity of proliferation-related, anti-apoptotic and pro-inflammatory genes, including TNF-α. Therefore, its malfunction affects many processes that may promote cancer formation (Concetti and Wilson 2018). It was observed that decreased miR-572 levels were associated with better overall survival in patients with renal cell carcinoma (Pan et al. 2018). Moreover, its knockdown inhibited proliferation and stimulated apoptosis of cancer cells (Guan et al. 2018). The high activity of miR-572 was associated with the promotion of migration and colorectal cancer invasion (Wang et al. 2018), as well as the Wilms’ tumor metastasis (Zhang et al. 2019, p. 1). The decrease in the expression of miR-572, accompanied by the overexpression of NFKB1 observed in our study, may indicate a different regulatory mechanism in endometrial cancer. The discussed miRNAs can act as a suppressor in the initial stage of endometrial cancer, which begins to gradually disappear with stimulation of the TNF-α/NF-κB axis.

TAB2 is also involved in the activation of NF-κB and TNF-α signaling. Its task is to bind transforming growth factor β-activated kinase 1 (TAK1), associated with the TNF receptor complex (Broglie et al. 2010, p. 1). In our study, the change in TAK1 level was not significant, whereas TAB2 was overexpressed, which could be related to the decrease in miR-1228-3p, miR-134-3p, miR-143-5p, miR-155-5p, and miR-22-5p expression. In a study by Xue et al., miR-1228-3p was upregulated in non-small cell lung cancer and associated with a bad prognosis (Xue et al. 2020). Similarly, in endometrial cancer, miR-1228-3p expression was higher than in the control, but the tested material was serum (Bloomfield et al. 2022). In turn, Zhao et al. showed that the high activity of miR-134-3p suppresses the progression of ovarian cancer (Zhao et al. 2021b). Moreover, overexpression of this miRNA in endometrial cancer inhibits its stem cells and thus the neoplastic process (Gao et al. 2015). Low levels of miR-143-5p can stimulate metastasis and the epithelial–mesenchymal transition (EMT) in gallbladder cancer (He et al. 2017) and breast cancer (Xu et al. 2022). In the case of miR-155-5p, Xu et al. described a decrease in its level in triple-negative breast cancer accompanied by an increase in the anti-cancer effect of cetuximab (Xu et al. 2021b). In turn, miR-29a-5p is considered a tumor suppressor, which was confirmed, among others, in gliomas (Dai et al. 2020), pancreatic cancer (Tréhoux et al. 2015), and hepatocellular carcinoma (Liang et al. 2018). Interestingly, Tokumaru et al. noted that the low activity of this miRNA affects the survival and aggressiveness of gastric cancer (Tokumaru et al. 2021).

The conducted analyses allowed to identify important genes related to TNF-α signaling, as well as miRNAs potentially involved in the regulation of their activity in endometrioid endometrial cancer. The microarray experiment was successfully validated with RT-qPCR and then with ELISA, which allowed for multilevel imaging of the expression profile of the studied genes. Additionally, miRNAs that may be subjected to more detailed analysis in future research have been proposed. The lack of such analysis in this study can be considered its weakness as miRNAs are identified based on an algorithm and not experimental data. Moreover, the study is also limited by a relatively small group of patients, so in the future, the obtained results should be validated on a larger cohort of patients.

TNF-α signaling in endometrial cancer is impaired, which worsens as the cancer progresses. This study indicates a significant role of the TNF-α/NF-κB axis in the course of endometrial cancer. The observed abnormalities may be the result of miRNAs’ activity in the initial stage of the disease and its loss as the cancer progresses.

## Methods

### Patients’ samples

The following study was approved by the Bioethical Committee operating at the Regional Medical Chamber in Krakow, no. 185/KBL/OIL/2020 and 186/KBL/OIL/2020, 20 September 2020. All procedures involving human participants were performed in accordance with the guidelines of the 2013 Declaration of Helsinki. The confidentiality of the data and the anonymity of the patients were maintained at all times. Informed consent was obtained from all participants involved in this study.

The study included patients who were qualified for hysterectomy. The study group consisted of 45 patients diagnosed with endometrioid endometrial cancer (EEC). The collected surgical samples were subjected to histopathological evaluation and divided into three subgroups according to the degree of histological differentiation: G1, 15 samples; G2, 15 samples; G3, 15 samples. The exclusion criteria were the diagnosis of non-endometrioid endometrial cancer, endometriosis, coexistence of another cancer, extreme obesity, and use of hormone therapy 24 months prior to surgery. The control group consisted of 45 patients without neoplastic changes who underwent surgery due to the prolapse of the uterus. The absence of cancer was also confirmed histopathologically. Patient characteristics are presented in Table 5.

**Table 5:** Characteristics of patients enrolled in the study

Group	C	G1	G2	G3
Age (years)	66.20 ± 4.34	65.67 ± 5.63	67.20 ± 3.53	68.13 ± 5.05
Height	1.60 ± 0.02	1.60 ± 0.03	1.59 ± 0.02	1.60 ± 0.03
Weight	73.49 ± 6.06	73.20 ± 6.54	75.27 ± 5.95	76.67 ± 4.67
BMI	28.79 ± 2.57	28.49 ± 2.32	29.61 ± 2.44	30.14 ± 1.96

*C* control, *G* grade of endometrial cancer

The collected tissue samples were placed in tubes containing Allprotect Tissue Reagent (Qiagen GmbH, Hilden, Germany, Cat No. 76405) and stored according to the manufacturer’s instructions. Total RNA was extracted using the TRIzol reagent (Invitrogen Life Technologies, Carlsbad, CA, USA, Cat No. 15596026). Its quality was assessed by electrophoresis (SUBMINI K. Kucharczyk T.E., Poland) and its quantity was evaluated by spectrophotometry (GeneQuant II spectrophotometer; Pharmacia LKB Biochrom Ltd., UK).

### mRNA microarrays

HG-U133A 2_0 oligonucleotide microarrays (Affymetrix, Santa Clara, CA, USA) and the GeneChip™ HT 3′IVT PLUS Reagent Kit (ThermoFisher, Waltham, MA, USA, Cat No. 902417) were used to determine the expression profile of genes involved in TNF-α signaling. 8 µg of RNA was used as a template to synthesize cDNA with SuperScript Choice System (Invitrogen Technologies, Carlsbad, CA, USA). BioArray HighYield RNA Transcript Labeling Kit (Enzo Life Sciences, Farmingdale, NY, USA) was then used to synthesize biotinylated cRNA. It was later purified with RNeasy Mini Kit (Qiagen GmbH, Hilden, Germany). The next step included fragmentation of the biotin-labeled cRNA with the Sample Cleanup Module Kit (Qiagen GmbH, Hilden, Germany). After hybridization to the microarray, cRNA was stained with streptavidin–phycoerythrin. A GeneArray scanner (Agilent Technologies, Santa Clara, CA, USA) was used to acquire the fluorescence signals. The list of genes related to TNF-α signaling was prepared based on data from the PathCards database (http://pathcards.genecards.org/) accessed on April 27, 2022 (Belinky et al. 2015).

### Real-time quantitative reverse transcription reaction (RT-qPCR)

The results obtained in the microarray analysis were further validated with RT-qPCR. The expression profile was determined for the TNF-α, TNFR1, TNFR2, CAV, NFKB1, and TAB2 genes using the SensiFast™ SYBR No-ROX One-Step Kit (Bioline, London, UK) and β-actin (ACTB) as endogenous control. The thermal profile included reverse transcription (45 °C, 10 min), polymerase activation (95 °C, 2 min), and 40 cycles of denaturation (95 °C, 5 s), annealing (60 °C, 10 s), and elongation (72 °C, 5 s).

For each run, a standard curve was plotted based on the β-actin quantitative standard (TaqMan DNA Template Reagent kit, Applied Biosystems, Foster City, CA, USA) at five different concentrations (400, 800, 2000, 4000, and 8000 copies of *ACTB* cDNA). Opticon™ DNA Engine Sequence Detector (MJ Research Inc., Watertown, MA, USA) calculated the mRNA copy numbers of studied genes in each sample. Table 6 lists the primer sequences.

**Table 6:** Primer sequences of the studied genes

mRNA	Sequence	Product size (bp)
*ACTB*	Forward 5′-TCACCCACACTGTGCCCATCTACGA-3′ Reverse 5′-CAGCGGAACCGCTCATTGCCAATGG-3′	295
*TNF-α*	Forward 5′-CTCTTCTGCCTGCTGCACTTTG-3′ Reverse 5′-ATGGGCTACAGGCTTGTCACTC-3′	135
*TNFR1*	Forward 5′-CCGCTTCAGAAAACCACCTCAG-3′ Reverse 5′-ATGCCGGTACTGGTTCTTCCTG-3′	134
*TNFR2*	Forward 5′-CGTTCTCCAACACGACTTCATCC-3′ Reverse 5′-ACGTGCAGACTGCATCCATGCT-3′	102
*CAV*	Forward 5′-CCAAGGAGATCGACCTGGTCAA-3′ Reverse 5′-GCCGTCAAAACTGTGTGTCCCT-3′	113
*NFKB1*	Forward 5′- GCAGCACTACTTCTTGACCACC-3′ Reverse 5′- TCTGCTCCTGAGCATTGACGTC-3′	130
*TAB2*	Forward 5′-TATTCAGCACCTCACGGACCCT-3′ Reverse 5′-CTTTGAAGTCGTTCCATTCTGGC-3′	141

*ACTB* β-actin, *TNF-α* tumor necrosis factor-alpha, *TNFR1* tumor necrosis factor receptor 1, *TNFR2* tumor necrosis factor receptor 2, *CAV1* caveolin 1, *NFKB1* nuclear factor kappa B subunit 1, *TAB2* TGF-beta activated kinase 1 (MAP3K7) binding protein 2, *bp* base pair

### Enzyme-linked immunosorbent assay (ELISA)

The expression profile of TNF, TNFR1, TNFR2, CAV1, NFKB1, and TAB2 proteins was assessed by ELISA according to the manufacturer’s instructions. The following kits were used in the study: Human TNF alpha ELISA Kit (Sigma-Aldrich, Saint Louis, MO, USA, Cat No. RAB1089), Human TNF-R1 ELISA Kit (MyBioSource, San Diego, CA, USA, Cat No. MBS167687), Human TNF-R2 ELISA Kit (MyBioSource, San Diego, CA, USA, Cat No. MBS7720492), Human Caveolin 1 ELISA Kit (MyBioSource, San Diego, CA, USA, Cat No. MBS727132), Human Nuclear Factor Kappa B (NFkB) ELISA Kit (MyBioSource, San Diego, CA, USA, Cat No. MBS450580), and Human TGF-beta-activated kinase 1 and MAP3K7-binding protein 2 ELISA Kit (MyBioSource, San Diego, CA, USA, Cat No. MBS762519).

### miRNA microarrays and miRNA target prediction

The miRNA expression profile was determined with miRNA 2.0 microarrays (Affymetrix, Inc., Santa Clara, CA, USA) according to the manufacturer’s protocol. GeneChip Scanner 3000 7G (Affymetrix, CA, USA) and Affymetrix GeneChip Command Console Software (AGCC) were used to read the obtained signals.

The mirDIP database was used to predict miRNAs targets among genes related to TNF-α signaling. The miRanda algorithm and bidirectional search with very high confidence filter were used (Tokar et al. 2018).

### Statistical analysis

Analysis of results from mRNA and miRNA microarray experiments was performed using the Transcriptome Analysis Console software (Thermo Fisher Scientific, Waltham, MA, USA). ANOVA and Tukey’s post hoc test were performed, and a Venn diagram was constructed (*p* < 0.05; FC > 2 or FC < − 2). Analysis of the RT-qPCR and ELISA results was performed on R using RStudio (version 4.2.0, RStudio, Inc.). The GEPIA database was used for overall survival analysis (http://gepia.cancer-pku.cn/).

## Data Availability

Data are included in the manuscript.
